# Engineered Porosity in Microcrystalline Diamond-Reinforced PLLA Composites: Effects of Particle Concentration on Thermal and Structural Properties

**DOI:** 10.3390/ma18194606

**Published:** 2025-10-04

**Authors:** Mateusz Ficek, Franciszek Skiba, Marcin Gnyba, Gabriel Strugała, Dominika Ferneza, Tomasz Seramak, Konrad Szustakiewicz, Robert Bogdanowicz

**Affiliations:** 1 Faculty of Electronics, Telecommunication and Informatics, Gdańsk University of Technology, Gabriela Narutowicza 11/12, 80-233 Gdańsk, Poland; matficek@pg.edu.pl (M.F.); franciszek.skiba@pg.edu.pl (F.S.); marcin.gnyba@pg.edu.pl (M.G.); 2 Department of Materials Science and Technology, Institute of Manufacturing and Materials Technology, Faculty of Mechanical Engineering and Ship Technology, Gdańsk University of Technology, 80-233 Gdańsk, Poland; gabriel.strugala@pg.edu.pl; 3 Department of Polymer Engineering and Technology, Faculty of Chemistry, Wrocław University of Science and Technology (WUST), Wyb. Wyspiańskiego 27, 50-370 Wroclaw, Polandkonrad.szustakiewicz@pwr.edu.pl (K.S.); 4 Department of Manufacturing and Production Engineering, Institute of Manufacturing and Materials Technology, Faculty of Mechanical Engineering and Ship Technology, Gdańsk University of Technology, 80-233 Gdańsk, Poland; tomasz.seramak@pg.edu.pl

**Keywords:** composites, PLLA, diamond, porosity, tomography, thermally induced phase separation

## Abstract

This research explores microcrystalline diamond particles in poly(L-lactic acid) matrices to create structured porous composites for advanced biodegradable materials. While nanodiamond–polymer composites are well-documented, microcrystalline diamond particles remain unexplored for controlling hierarchical porosity in systems required by tissue engineering, thermal management, and filtration industries. We investigate diamond–polymer composites with concentrations from 5 to 75 wt% using freeze-drying methodology, employing two particle sizes: 0.125 μm and 1.00 μm diameter particles. Systematic porosity control ranges from 11.4% to 32.8%, with smaller particles demonstrating reduction from 27.3% at 5 wt% to 11.4% at 75 wt% loading. Characterization through infrared spectroscopy, X-ray computed microtomography, and Raman analysis confirms purely physical diamond–polymer interactions without chemical bonding, validated by characteristic diamond lattice vibrations at 1332 cm^−1^. Thermal analysis reveals modified crystallization behavior with decreased melting temperatures from 180 to 181 °C to 172 °C. The investigation demonstrates a controllable transition from large-volume interconnected pores to numerous small-volume closed pores with increasing diamond content. These composites provide a quantitative framework for designing hierarchical structures applicable to tissue engineering scaffolds, thermal management systems, and specialized filtration technologies requiring biodegradable materials with engineered porosity and enhanced thermal conductivity.

## 1. Introduction

Recent advances in nanomaterial–polymer composites have expanded applications across construction, medicine, protection, and energy sectors. Particularly, diamond particles have emerged as promising fillers due to their exceptional optical, thermal, chemical, and mechanical properties. They exhibit broad optical transparency from ~225 nm to the far infrared, high thermal conductivity (~2200 W·m^−1^·K^−1^ at room temperature), and outstanding chemical inertness, remaining unaffected by acids and other chemicals. This chemical stability, combined with their biocompatibility, makes them suitable for biomedical applications [1]. Diamond has an exceptionally high Young’s modulus in the range of 1050–1200 GPa, depending on crystal orientation [2]. Due to these properties, the incorporation of diamond nanoparticles into polymeric materials has opened novel application domains, yet several challenges persist regarding particle dispersion, interfacial adhesion, and cost-effective fabrication methods [3].

Existing literature demonstrates the significant potential of nanodiamond–polymer composites [4]. Rheman et al. [5], in a review paper, highlighted how nanodiamond integration with fibrous substrates enhances mechanical and thermal stability, though noting challenges with particle aggregation due to Van der Waals and electrostatic forces. For instance [6], poly(lactic acid) (PLA) nanofibers incorporating 1 wt% nanodiamonds exhibited an increase in tensile strength from 0.33 MPa to 1.12 MPa, an improvement in Young’s modulus from 4.10 MPa to 9.95 MPa, an increase in elongation at break from 23.02% to 56.82%, and an enhancement in fracture toughness from 0.08 MPa·m^1/2^ to 0.46 MPa·m^1/2,^ relative to neat PLA nanofibers. Zhao et al. [7] reported substantial improvements in mechanical properties and thermal stability of PLA through nanodiamond incorporation, with storage modulus increased by 75% for 3 wt% nanodiamond, reaching 0.7 GPa at 130 °C, and thermal decomposition delayed by 10.1 °C, increasing from 313.9 °C for neat PLA to 324.0 °C with the addition of 1 wt% nanodiamond at minimal loadings. These improvements were attributed to homogeneous nanocluster dispersion and unique bridge morphologies between the PLA matrix and diamond particles. Recent work has also addressed PLA’s inherent brittleness through diamond functionalization strategies. Researchers [8] combined thermoplastic polyurethane with curcumin-functionalized nanodiamonds, where the incorporation of 1.5 wt% nanodiamond resulted in an elongation at break of 124.8%, corresponding to a 119.9% increase compared to the PLA sample and a 47.9% improvement in thermal stability (T_5%_), with the decomposition temperature increasing from 225.2 °C for neat PLA to 333.0 °C for the material with 1.5 wt% nanodiamond content. Han et al. [9] further demonstrated simultaneous enhancement of strength and fracture toughness through maleic anhydride functionalization, with improvements at 1.5 wt% nanodiamond content compared to neat PLA: a 41.0% increase in tensile strength (from 40.7 MPa to 57.3 MPa), a 50.6% increase in tensile modulus (from 1691.9 MPa to 2548.6 MPa), and a 543.7% increase in elongation at break (from 19.6% to 126.0%). Recent advances in PLA-based composites demonstrate significant thermal property enhancement through strategic diamond filler incorporation and interfacial engineering. Surface modification of microdiamond particles with octadecylamine (ODA) establishes hydrogen bonding interactions with hydroxyl groups, creating a PLA/ODA/microdiamond interfacial architecture that achieves thermal conductivity values of 2.22 W m^−1^ K^−1^—representing approximately a ten-fold enhancement over pristine PLA [10]. The hydroxyl-terminated diamond surface [11] has been reported as beneficial for silanization processes, allowing for optimization of surface functionalization protocols and presenting a viable pathway to enhance interfacial bonding characteristics.

Systematic investigations across nano/micro-diamond concentration ranges reveal optimal performance at 1–5 wt% loadings, which simultaneously enhance tensile modulus from 0.93 GPa to 1.34 GPa, crystallization kinetics, and thermal stability, while higher concentrations (10 wt%) exhibit diminished properties due to agglomeration effects [12].

Further developments by Zhang et al. [13] demonstrated enhanced membrane performance through deep eutectic solvent-modified nanodiamond addition, particularly improving hydrophilicity, porosity, and antifouling properties in polyimide ultrafiltration membranes. Similarly, Siddiqa et al. [14] achieved enhanced antifouling performance in polyaniline/polyvinylidene fluoride composite membranes containing nanodiamonds, reaching fouling recovery ratios up to 88%. Idbal et al. [15] expanded applications into food packaging by developing polyvinyl alcohol-nanodiamond composites with enhanced thermal stability, mechanical properties (tensile strength ~60 MPa, ductility ~180%), UV protection, and antibacterial activities against E. coli and S. aureus. Cieślik et al. [16] pioneered 3D-printable PLA composites containing detonation nanodiamonds and boron-doped carbon nanowalls for electroanalytical applications, achieving enhanced redox process kinetics and lower detection limits for dopamine compared to carbon black-loaded PLA. These findings establish diamond-filled PLA composites as promising thermal management materials for electronic applications, with interfacial compatibility serving as the critical determinant of composite performance optimization.

The current research landscape predominantly focuses on detonation nanodiamonds, which differ significantly from microcrystalline particles synthesized via high-pressure high-temperature (HPHT) methods [17,18]. This critical knowledge gap presents an opportunity for novel exploration into larger-scale diamond particle effects within polymer matrices. The main research objective of our research was to introduce a paradigm shift by incorporating microcrystalline diamond microparticles into a highly porous PLA matrix to form multi-hierarchical composites—a challenge that has remained a significant research problem to date. Unlike previous studies utilizing nanoscale diamond particles, our approach leverages microcrystalline diamond particles synthesized via HPHT, offering distinct size-dependent properties and surface chemistry. In our state-of-the-art development, by controlling the weight ratio of diamond in PLLA from 5 to 75 wt%, we achieve unprecedented control over hierarchical structure formation across multiple length scales. This technique creates extraordinarily porous materials with complex architecture that promotes unique thermal behaviors not achievable with conventional nanodiamond composites. Through comprehensive characterization employing FTIR spectroscopy, X-ray tomography, Raman analysis, and thermal techniques (TGA, DSC), we demonstrate superior crystallinity control and thermal stability. Given the scarcity of comparative analyses of spatial porosity in the literature, this study applies X-ray tomography to PLLA samples to provide a detailed assessment of micro- and nanodiamond contents as modifiers, yielding novel insights into pore formation and its influence on thermal properties. The resulting multi-hierarchical structures represent a significant advancement in biodegradable polymer composites with potential applications spanning tissue engineering, thermal management systems, and specialized filtration technologies where nanodiamond composites have shown limitations.

## 2. Experimental Set-Up

### 2.1. Materials

Poly(L-lactide), (Resomer L210s, PLLA) from Evonik, Hanau, Germany, having density of d = 1.25 g/cm^3^ was used as a matrix and 1,4-dioxane from Eurochem BGD, Poland, was used as a solvent. Diamond microparticles (MDPs) produced by HPHT (high-pressure, high-temperature) procedure were commercially obtained from Pureon AG (Switzerland; ‘MSY 0–0.25 and MSY 0.75–1.25′) and supplied in powder form. The diamonds were not further modified or functionalized. A MDP has an oriented crystal structure with parallel cleavage planes, similar to natural diamond structures. Details about MDPs (Pureon, Lengwil TG, Switzerland) are provided in Table 1.

### 2.2. Fabrication Procedure of PLLA/MDP Composites

In this work, a number of experimental materials were produced. Because the research is pioneering, we decided to create as many composites as possible with varying compositions, with diamond additives ranging from 0 to 75% by weight. PLLA was chosen because it is a thermoplastic polymer with well-known properties, is commercially available, and commonly used to produce porous structures using the Thermally Induced Phase Separation technique. Due to its application potential, we wanted the bioactive diamond to be dispersed over the largest possible surface area; hence, we chose a foamed polymer with diamond particles. The 2.5 wt% solution of PLLA in 1,4-dioxane was prepared as follows: a PLLA sample was weighed (with an accuracy of ±0.001 g) and the 1,4-dioxane was weighted (with an accuracy of ±0.01 g). The weighed PLLA samples were introduced into the glass bottle with 1,4-dioxane. The bottle was capped with a screw cap and tightly wrapped with Parafilm. The mixture thus prepared was left to stir for 24 h to dissolve PLLA in 1,4-dioxane on a magnetic stirrer at T = 60 °C, with the stirrer speed f = 500 rpm.

Then, 7 g of each solution was taken and poured into 6 smaller vessels into which the diamond was then weighed (to an accuracy of ±0.0001 g). Next, MDPs were added to the PLLA/1,4-dioxane solution in such a way that the weight ratio of PLLA to diamond was 95/5 to 25/75. The weight ratio of diamond in PLLA from 5 to 75 wt% was used. Higher concentration causes stability problems. All the obtained composite samples are listed in Table 2. The samples were left on a magnetic stirrer for 24 h.

**Figure 1:**
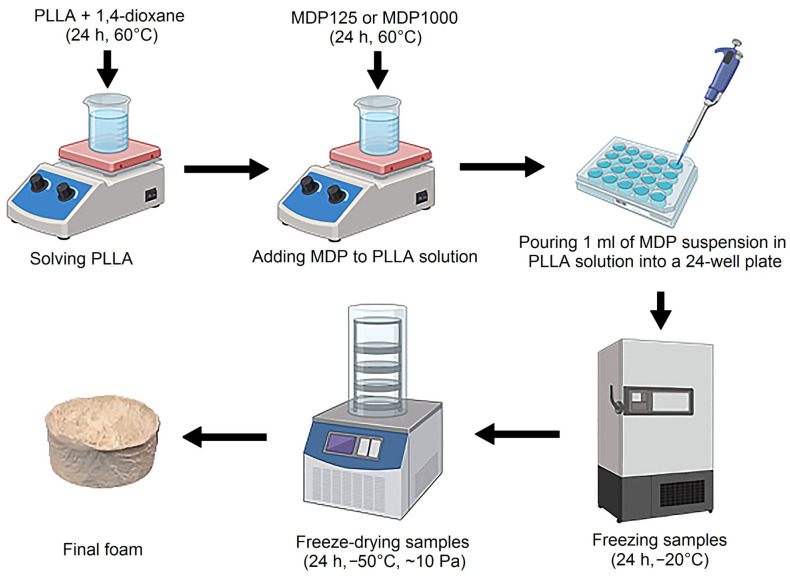
Scheme of PLLA–MDP composite fabrication [19].

After that, the suspension of diamond powder in PLLA/1,4-dioxane solutions were introduced into the 24-well plate (1 mL to one well). Samples were frozen for 24 h prior to lyophilization. Then, the samples were moved to a freeze dryer (Labconco 2.5, Kansas City, USA) for 24 h, at a temperature of −50 °C and a pressure of ~20 Pa. This process yielded foam composites with varying MDP loadings. The reference sample was prepared without MPD addition (see Figure 1).

### 2.3. Structural and Thermal Characterization of PLLA/MDP Composites

Attenuated Total Reflectance–Fourier Transform Infrared Reflectance (ATR-FTIR) spectra were recorded in ATR mode using a Nicolet iZ10 spectrometer (Thermo Scientific, Waltham, MA, USA) over the spectral range of 600–4000 cm^−1^. Each spectrum was acquired by averaging 32 scans at a resolution of 4 cm^−1^.

The molecular composition of the samples was investigated by means of Raman spectroscopy using Raman confocal microscope (Horiba LabRAM ARAMIS, Kyoto, Japan). Spectra were recorded in a range of 200–3500 cm^−1^ with an integration time of 60 s (3 averages), using a 532 nm diode pumped solid state (DPSS) laser in combination with a 50 × objective magnification (NA = 0.5) and a 185 μm confocal aperture. The spectra were recorded in a few different places on the samples because of their porosity and expected inhomogeneity. Raman spectra were subsequently processed with use of OriginPro 2020 (OriginLab, Northampton, MA, USA).

Cross-sections at the center of each sample were made using a razor blade for microscopic examination. The surfaces of the cross-sections were examined using a scanning electron microscope (SEM, JEOL JSM-7800F, Tokyo, Japan) operating in Gentle Beam mode with an accelerating voltage of 0.5 kV. An in-lens detector was used to capture both secondary and backscattered electrons.

A GE phoenix vǀtomeǀxs computed microtomograph (µCT, Waygate Technologies, Wunstorf, Germany) was used to perform 3D scans. The X-ray power was set at 21.45 W, with a voltage of 65 kV and a current of 330 μA. Each radiograph was captured with an exposure time of 5000 ms. To improve image quality, two copper filters, each 0.1 mm thick, were used. The 3D scans were performed with a voxel resolution of 19.64 µm for all samples. During a full 360-degree rotation of the sample, 1500 radiographs were collected. Each radiograph represented the average of fifteen individual projections to enhance the signal-to-noise ratio. The 3D reconstruction process was carried out using Phoenix Datos/2 software, applying a low-noise reconstruction algorithm. Porosity analysis was performed in VGStudio Max 2.2, using a threshold-based algorithm to calculate porosity. For each sample, the region of interest (ROI) was defined as the cylindrical volume within the sample. The porosity was calculated by determining the ratio of pore volume to the ROI volume, which included both the solid material and the pores.

The composites porosity test was performed by measuring the apparent density of the samples on a hydrostatic balance H-300S (Hildebrand Electronic Densimeter, Wendlingen am Neckar, Germany), having an accuracy of ±0.001 g. The measurement was repeated three times for each material. The porosity was calculated from Equation (1):

(1) $$\Phi =\left[1-\frac{\rho_{p}}{\rho_{l}}\right]$$

where *ϕ*—porosity, *ρ_p_*—apparent density of the foam composite, *ρ_l_*—true density of bulky composite.

Representative samples for DSC analysis were taken from the center part of the foams. Analysis was performed using a DSC1 Mettler Toledo (Mettler Toledo, Greifensee, Switzerland) differential microcalorimeter, with temperatures ranging from 25 °C to 200 °C, at a rate of 5 K/min, in a nitrogen atmosphere. Evaluations of the results were performed in the STARe Evaluation Software 19.00, with the glass transition temperatures (Tg) calculated using the first derivative with 50 smoothing points. The mass-normalized data were also exported as txt files to Origin 2024b for presentation of the results.

A Mettler Toledo TGA/DSC1 (Mettler Toledo, Greifensee, Switzerland) was used for the thermal analysis. Measurements were performed in the temperature range from 25 °C to 900 °C, with a heating rate of 10 K/min, in a nitrogen atmosphere. Evaluations of the results were performed in the STARe Evaluation Software, with the temperature values Td,max [°C] calculated from the first derivative with 50 smoothing points. The TGA data were exported as CSV files to Origin 2024b for presentation of the results.

## 3. Results and Discussion

### 3.1. Topography and Structure of PLLA/MDP Composites

**Figure 2:**
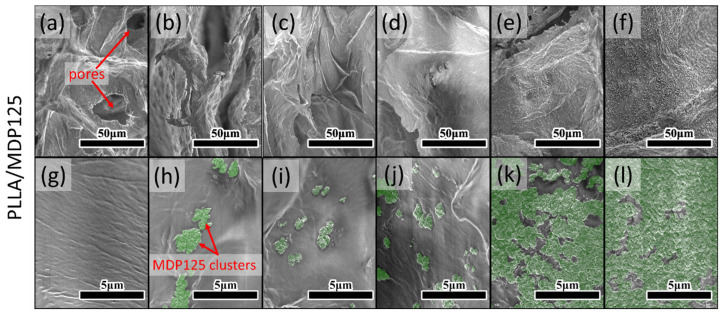
SEM images of cross-sections for samples with MDP125 powder. Images (**a**–**f**) present a general overview. Images (**g**–**l**) present higher magnification with clearly visible, highlighted diamond nanoparticles. The ratio of diamond particles to PLA increases from left to right: (**a**,**g**) PLLA, (**b**,**h**) PLLA/MDP125-5, (**c**,**i**) PLLA/MDP125-10, (**d**,**j**) PLLA/MDP125-15, (**e**,**k**) PLLA/MDP125-50, (**f**,**l**) PLLA/MDP125-75.

PLLA/MDP sample surface images made with a scanning electron microscope (SEM) are displayed in Figure 2. The SEM images of different PLA to ND ratios, from pure PLA up to 75% of diamond powder, are presented in Figure 2, with green highlighted diamond particles. The general structure can be characterized as consisting of many packed, intersecting layers and faces of polymer matrix. It is also visible that the surface development decreases as the amount of powder increases, which is further confirmed with 3D reconstructions. At lower diamond contents, small agglomerates of 125 nm nanoparticles are created, as mentioned by Zhao et al. [7], and this tendency persists for samples PLLA/MDP125-5, PLLA/MDP125-10, and PLLA/MDP125-15. As the amount of diamond powder increases, so does the number of agglomerates, and their size varies between 0.5 and 1.5 µm. For the two highest concentrations, the surface is either almost fully or fully covered by a uniform diamond layer. The coverage of polymer is presented in Table S1.

**Figure 3:**
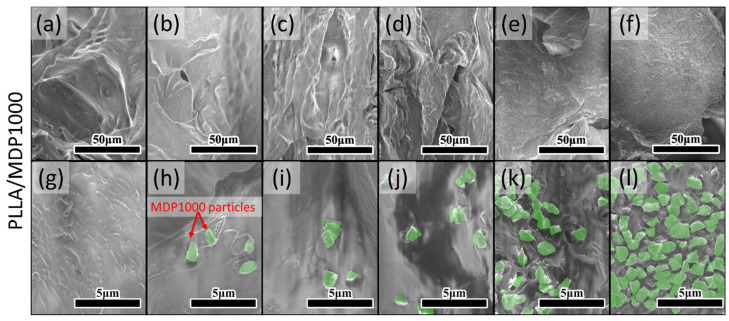
SEM images of cross-sections for samples with MDP1000 powder. Images (**a**–**f**) present a general overview. Images (**g**–**l**) present higher magnification with clearly visible, highlighted diamond nanoparticles. The ratio of diamond particles to PLA increase from left to right: (**a**,**g**) PLLA, (**b**,**h**) PLLA/MDP1000-5, (**c**,**i**) PLLA/MDP1000-10, (**d**,**j**) PLLA/MDP1000-15, (**e**,**k**) PLLA/MDP1000-50, (**f**,**l**) PLLA/MDP1000-75.

The SEM images of samples synthesized with MDP1000 are presented in Figure 3. The lower magnification images presented in Figure 3a–f have similar characteristics to those with MDP125. The polymer matrix is wrinkled and coiled, which creates many pores and complex surfaces. The complexity of the surface also decreased with addition of diamond particles, and this general trend is also supported by 3D reconstructions. However, in opposition to MDP125 powder, the bigger diamond particles do not tend to agglomerate, but spread individually across the surface [20], which is true even for the highest presented ratio of diamond powder. The diamonds are not as closely packed as the MDP125, leaving small spaces between individual grains, which also results in lower total coverage in comparison to sample the corresponding sample PLLA/MDP125-75 with the same ratio of PLLA to diamond powder, as presented in Table S1.

**Figure 4:**
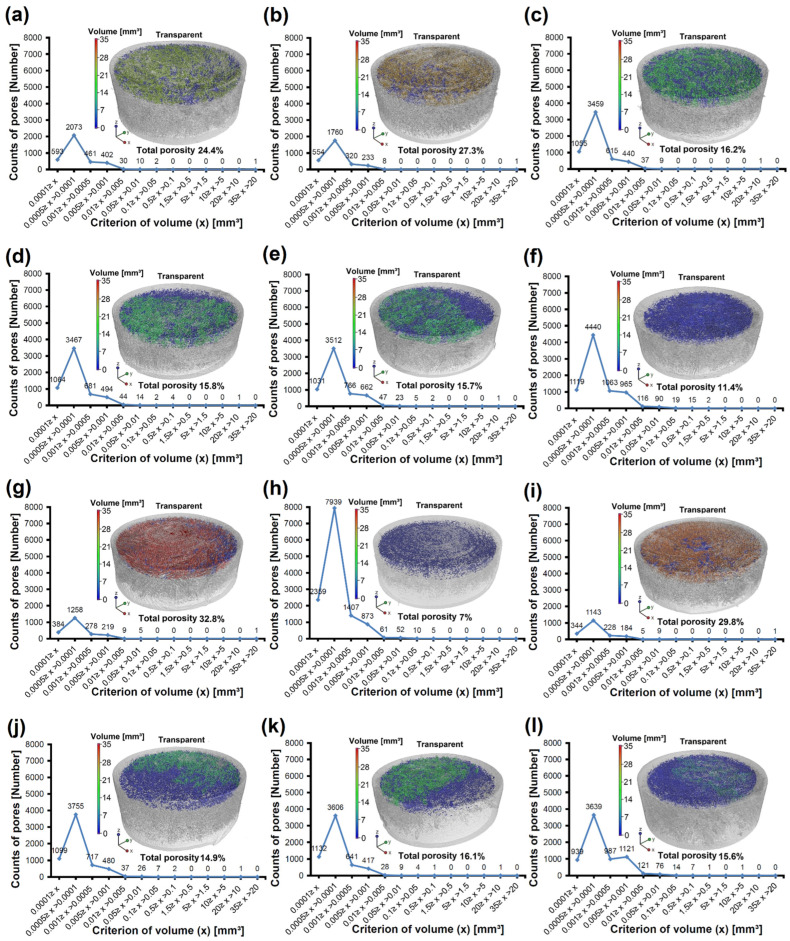
Three-dimensional reconstructions of samples with solid material visualization at 50% transparency, volumetric pore quantification and pore size distribution graphs as a function of volume: (**a**) PLLA, (**b**) PLLA/MDP125-5, (**c**) PLLA/MDP125-10, (**d**) PLLA/MDP125-15, (**e**) PLLA/MDP125-50, (**f**) PLLA/MDP125-75, (**g**) PLLA, (**h**) PLLA/MDP1000-5, (**i**) PLLA/MDP1000-10, (**j**) PLLA/MDP1000-15, (**k**) PLLA/MDP1000-50, (**l**) PLLA/MDP1000-75.

The 3D reconstructions were performed to verify the spatial porosity of samples containing two types of diamonds (MDP125 and MDP1000) with varying contents in the base material, which was PLLA. The results of these analyses for samples ranging from neat PLLA up to 75% MDP125 content in PLLA are presented in Figure 4a–f. It was observed that the unmodified sample shown in Figure 4a exhibited a porosity of 24.4%. In the case of the sample with the lowest MDP125 diamond content (5%) presented in Figure 4b, the porosity increased to 27.3%. Samples with diamond contents ranging from 10% to 50% MDP125 demonstrated a porosity of approximately 16%, as shown in Figure 4c–e. The lowest porosity of 11.4% was recorded for the sample with the highest MDP125 diamond content (75%), as shown in Figure 4f. For each of the analyzed samples, pore size distributions were calculated and depicted in graphs in Figure 4a–f. It was determined that as the MDP125 content in PLLA increased, the proportion of a single large-volume pore decreased, resulting in lower overall spatial porosity. However, it should be noted that the single large-volume pore actually constituted spatially connected small pores, which was illustrated in Figure 4 by using different colors to indicate pore volumes. For example, in neat PLLA, the largest pore was marked in yellow, as shown in Figure 4a. Additionally, it was observed that as the content of MDP125 in PLLA increased, the number of small-volume pores increased, which can be considered as closed pores. These pores were visualized in Figure 4 with blue coloring on partially transparent 3D visualizations. As a result of modification with MDP125 diamonds, the total porosity of the samples decreased, showing a downward trend as the MDP125 content increased.

For the second set of samples, related to MDP1000 diamonds, the reference sample presented in Figure 4g was another neat PLLA sample with a porosity of 32.8%—the highest porosity value among all analyzed samples (MDP125 and MDP1000). The differences in the total pore volume of the reference samples (neat PLLAs) are due to the production method illustrated in Figure 1. During the injection of liquid PLLA (prior to polymerization) into circular molds, turbulent flow occurs, leading to the formation of an irregular structure of a single large pore. This large pore, formed during the injection and solidification of PLLA into foam, is responsible for the observed differences in porosity between the reference samples. Such behavior is typical of polymer-based samples produced by manual injections. Therefore, two sets of samples were compared, including a reference sample and all diamond contents (MDP125 and MDP1000). Both sets were fabricated sequentially. In the case of the sample with the lowest MDP1000 content (5%) presented in Figure 4h, the porosity significantly decreased to 7%. Such a low porosity value should be interpreted as an abnormality of unspecified origin. For the sample with 10% MDP1000 content shown in Figure 4i, the porosity was 29.8%, which was lower than that of the reference sample (Figure 4g). Subsequently, for MDP1000 contents ranging from 15% to 75%, the porosity ranged from just under 15% to slightly over 16%, as presented in Figure 4j–l. Moreover, the pore distribution shown in the graphs in Figure 4g–l exhibited a similar trend as observed in the samples containing MDP125—high porosity was associated with a single large-volume pore, whose significance diminished with the increasing content of MDP1000, giving way to more numerous, small-volume closed pores. This phenomenon was illustrated as a color gradient in Figure 4g–l. It could be concluded that the modification of PLLA with both types of diamonds (MDP125 and MDP1000) affected the polymerization process, resulting in reduced total porosity and altered pore characteristics within the samples. This effect was particularly pronounced in PLLA samples with the highest diamond content (75%). Detailed values of the porosity results for all analyzed samples are provided in the Supplementary Information in Table S2.

**Figure 5:**
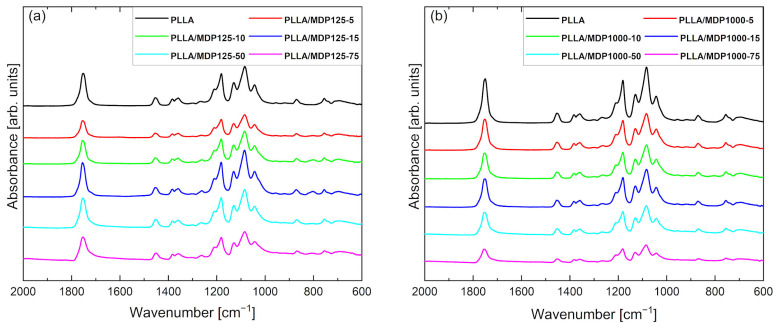
FTIR/ATR spectra of PLLA doped with (**a**) MDP125 and (**b**) MDP1000 samples. Lack of presence of new peaks indicates that no chemical interaction between polymer matrix and MDP occurs.

Figure 5 contains the FTIR/ATR curves for PLLA and composites doped with diamonds MDP125 (Figure 5a) and MDP1000 (Figure 5b). The FTIR/ATR curve for pure PLLA shows characteristic peaks and regions corresponding to specific structures and morphology of the composite. The peak at 694 cm^−1^ corresponds to C-H stretching and bending vibrations, while the peak at 757 cm^−1^ corresponds to C-H stretching and deformation vibrations associated with the crystal structure of the sample. The peak at 870 cm^−1^ corresponds to C-C stretching vibrations characteristic of esters in the C-COO group. The peaks at 1043, 1080, 1129, 1181, and 1210 cm^−1^ are associated with C-O stretching vibrations. Next, the peaks at 1358, 1383, and 1455 cm^−1^ correspond to C-H deformation vibrations. A distinct peak characteristic of esters at 1754 cm^−1^ is associated with the stretching of C=O in the carbonyl group [21]. No new peaks are observed in the FTIR spectra for composite foams regardless of the type of diamond, which indicates that there is no chemical interaction between the polymer matrix and the diamond powder. The lower intensity and peak shifts observed for some spectra are caused by the presence of different crystal structures in foams, directly related to PLLA polymorphism, as described in the literature [22]. The addition of diamond powders to the PLLA foam influences the presence of different PLLA crystal structures. Furthermore, it was found that for foams containing MDP1000 diamond powders, the decrease in peak intensity with increasing diamond content in the foam is also caused by the increasing powder content and grain size [23]. For the spectrum for foams with MDP125 (Figure 5a) diamond, the decrease in intensity does not show a dependence on the increasing diamond powder content, which means that the change in peak intensity in the ATR-FTIR spectrum for this series of foams is caused solely by PLLA polymorphism. As indicated by FTIR studies, among others, there is no reaction between the matrix and the filler. The diamond was dispersed in a PLLA solution in dioxane and, after freezing and freeze-drying, remained inside (occluded within the material).

**Figure 6:**
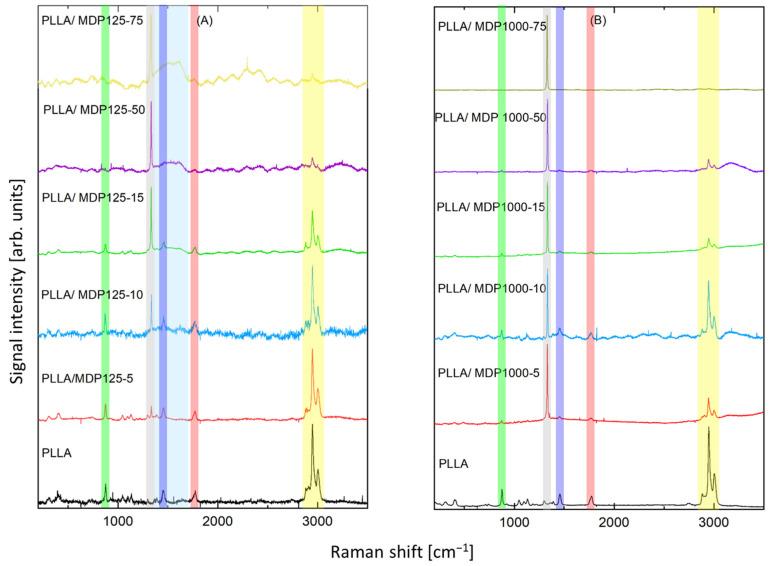
Raman spectra of two reference samples (neat PLLA) and all diamond contents of (**A**) MDP125 and (**B**) MDP1000 in PLLA. The colored rectangles, as described in the main text, mark the main Raman bands. (Attn. green: ν(C–COO) stretching mode, grey: diamond lattice oscillations, dark blue: δ_as_(C–H) asymmetric bending deformation, light blue: D and G band of non-diamond carbon phase, red: ν(C=O) stretching vibration, yellow: ν(C-H) stretching vibrations).

Raman spectra of the samples are shown in Figure 6. The dominating band at 1332 cm^−1^ (marked gray in Figure 6), which can be seen both in part A (smaller diamond crystals) and B (larger diamond crystals), is assigned to diamond lattice oscillations [24]. Its intensity growth with increasing content of diamond with subsequent decrease in the bands assigned to PLLA confirms increasing content of diamond in respective samples. In part A, two additional bands at about 1341- 1344 cm^−1^ and 1583-1587 cm^−1^ (marked light blue) appear. They are assigned to the “D” band and “G” band, respectively. Their presence confirms small content non-diamond carbon phases, which are typical to microdiamond and especially to nanodiamond. They were observed also in pure nanodiamond material. No additional impact of PLLA on the content of non-diamond carbon phases was noticed. A full set of the spectral lines characteristic of PLLA [16] was recorded, thus confirming the proper composition of the material. The strongest set of bands at 2884 cm^−1^, 2909 cm^−1^, 2949 cm^−1^, 3004 cm^−1^ (marked yellow) can be assigned to ν(C-H) stretching vibrations, such as ν(CH), ν_as_(CH_3_), and ν_s_(CH_3_). A strong band at 1774 cm^−1^ (marked red) is due to ν(C=O) stretching vibration.

Band 1455 cm^−1^ (marked dark blue) is assigned to δ_as_(C–H) asymmetric bending deformation, while the doublet of weak band at 1365 cm^−1^ and band at 1392 cm^−1^ is due to δ(CH_3_) symmetric bending mode. The band at 1298 cm^−1^ is due to δ_2_CH modes. The bands at 1225 cm^−1^ and 1184 cm^−1^ can be assigned to the C–O–C stretching modes of ester groups as asymmetric bands. The band at about 1096 cm^−1^ is ascribed to the symmetric vibrations of the ν_s_(COC) mode. At the same time, the band at about 1059 cm^−1^ is due to the ν(COC) modes. The bands at about 1046 cm^−1^ can be assigned to the ν(C–CH_3_) stretching mode. The weak band at 924 cm^−1^ is one of the characteristic vibrations of the helical backbone mixed with the CH_3_ rocking modes. The strong bands at 876 cm^−1^ (marked green) are due to the ν(C–COO) stretching mode of the repeat unit. The band observed at 739 cm^−1^ is mainly due to the δ(C–O) in-plane bending. The 713 and 679 cm^−1^ bands can be assigned to γ(C–O) out-of-plane bending. The weak bands at 583 and 515 cm^−1^ are mainly assigned to the δ_1_(C–CH_3_) and δ(CCO) bending. The strong asymmetric Raman doublet 398–413 cm^−1^ can be associated with δ(CCO) deformation. The medium-intensity band at 306 cm^−1^ is assigned to δ_s_(C–CH_3_) bending, while the weak shoulder at 347 cm^−1^ is due to δ(2C–CH_3_) Raman bands, respectively. The bands at 241 and 208 cm^−1^ correspond to CH_3_ torsion modes. Relative intensity of the bands assigned to PLLA decreases with growth of diamonds content. However, the main bands can be noticed in all samples, and the ratio of the main bands remained constant, confirming that addition of diamond was efficient and the assumed content was achieved while polymer was not damaged during addition of diamonds. Some spectra contained fluorescence signals or higher noise levels, which were a consequence of slightly different levels of Raman signals. These phenomena were a consequence of the impact of porous structure of the samples on Raman measurements.

### 3.2. Thermogravimetric and DSC Analyses of PLLA/MDP Composites

**Figure 7:**
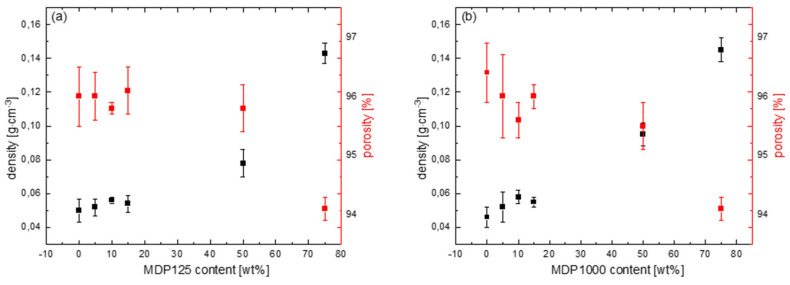
Effect of (**a**) MDP125 and (**b**) MDP1000 content on density (left black scale) and porosity (right red scale) of PLLA/MDP125 and PLLA/MDP1000 composites. The increase in density is insignificant even at high diamond loads and porosity decreases with increased diamond content.

Figure 7 shows the effect of MDP125 and MDP1000 diamond content on the porosity and density of foamed PLLA/MDP125 and PLLA/MDP1000 composite systems. Additionally, detailed numerical data on porosity and density measurements for PLLA samples containing all MDP125 and MDP1000 contents are presented in Tables S3 and S4 of the Supplementary Information, respectively. The PLLA reference sample has a porosity of 96% and a density of 0.046 g·cm^−3^. The addition of diamond with a density of ~3.5 g·cm^−3^ [25] affects the increase in density of the systems. At the same time, given the high porosity of the system, the low mass, and the fact that the diamond is located only in the walls, which are a few percent of the volume, the increase in density is not significant even with a high diamond content in the system.

For both diamonds sizes, the trends are identical—an increase in the diamond content of the system results in an increase in density and a decrease in porosity. The addition of MDP125, as well as MDP1000 at 5, 10, and 15 wt% levels, does not actually affect the density, or the porosity of the systems; the measured changes are all within the measurement errors. Visible changes can be observed at 50% addition, where for MDP125 the density is ~0.078 g·cm^−3^ at 95.8% porosity, and for MDP1000 the density is 0.095 g·cm^−3^ at 95.5% porosity. The largest changes were observed for systems containing 75% diamond, where the porosity for both MDP125 and MDP1000 is ~94% and the density is 0.143–0.145 g·cm^−3^.

**Figure 8:**
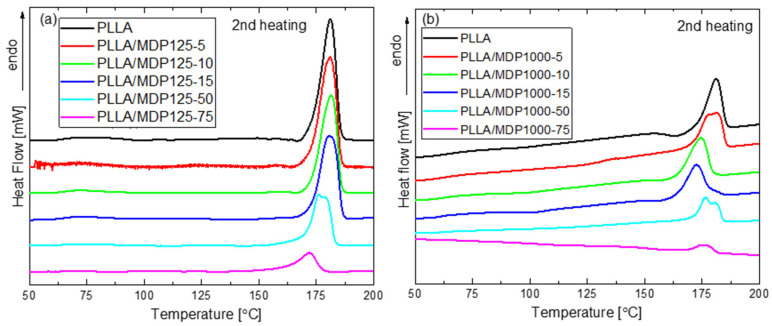
Second heating scan on DSC for PLLAs with (**a**) MDP125 and (**b**) MDP1000 series showing decreasing melting temperature with increased diamond loading.

Figure 8 shows the second scan of DSC heating of foamed PLLA-based composites and the MDP125 or MDP1000 diamonds. Figure 8a shows systems containing smaller diamonds (MDP125), while Figure 8b compares the plots of systems containing larger diamonds (MDP1000). Additionally, detailed DSC parameter values for PLLA samples containing all MDP125 and MDP1000 contents are provided in Table S5 and Table S6 of the Supplementary Information, respectively.

Analysis of the measured data indicates that the addition of diamond affects both the melting point of PLLA in the system and the enthalpy of melting. For PLLA doped with MDP125 in the range of 5–15 wt%, a melting point in the range of 180–181 °C was observed, while with a higher proportion of MDP125, the melting point decreased to 176 °C and 172 °C for systems containing 50 and 75 wt% MDP125 diamond, respectively.

At the same time, for an increase in MDP125 content, the enthalpy of melting decreases and is ~46 J/g for PLLA, while for the system containing 75 wt% MDP125, the enthalpy value decreased to ~14.5 J/g. In addition, an exothermic effect was observed before the onset of melting for the curves measured for PLLA and systems with low MDP125 content. According to the literature, this is an α`- α transition. In addition, a split peak was observed for the PLLA/MDP125-50 composite, indicating the presence of crystallites with different melting points [26].

For PLLA composites doped with MDP1000, essentially analogous trends were observed as for the MDP125 additive. The main difference is that in the case of the MDP1000 additive, a temperature reduction is observed already at 5 wt% of its content, and for each subsequent measurement point, the value gradually decreases up to ~175 °C for the system containing 75 wt% of this filler.

**Figure 9:**
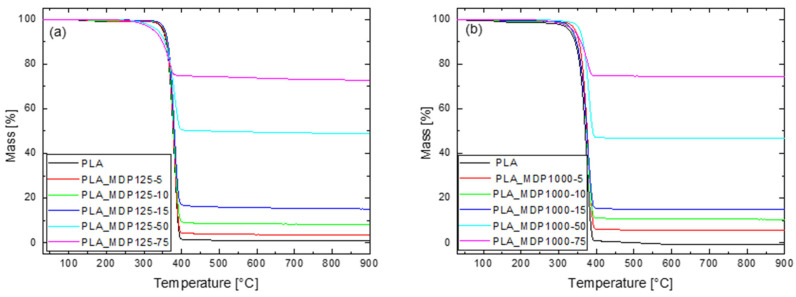
TGA curves for PLLA-based systems containing (**a**) MDP125 and (**b**) MDP1000 particles.

Figure 9 shows the TGA curves for the tested materials. Additionally, Tables S7 and S8 in the Supplementary Information present the TGA parameter values for PLLA samples with all MDP125 and MDP1000 contents, respectively. The results of the pyrolysis residue at 900 °C indicate that the residue values are close to the diamond content of the system. For example, for a system consisting only of PLLA, the mass loss at 900 °C is close to 100%, while for systems containing 75% diamond additives, the loss at 900 °C is close to 25%, which is the PLLA content of the system. In addition, for both MDP125 and MDP1000, the curves at analogous diamond content are close to each other, indicating that the behavior of the two types of additives is similar, and that the actual content of the additives in the system is very close to the theoretical assumptions.

The increase in diamond content leads to a reduction in the thermal stability of the PLLA matrix, as evidenced by the decrease in T_-5%_, Td, and T_d,max_ values. The reduction in T-5% values becomes evident at diamond contents exceeding 50 wt% in the PLLA/MDP125 foam. The residual masses (m580, m880) increase proportionally with the diamond content, confirming a strong consistency between the experimental data and the theoretical composition of the systems. This behavior can be attributed to the exceptional thermal conductivity of diamond, which accelerates the degradation of the polymer matrix, while the diamond itself remains thermally stable across the entire investigated temperature range. For PLLA/MDP1000 foams (Table S8) at low diamond contents (5–15%), a reduction in thermal stability is observed, with lower T-5%, Td, and T_d,max_ values, indicating that degradation occurs earlier. At 50% diamond content, an improvement in T_-5%_ and T_d,max_ is noted compared to samples with lower filler levels, suggesting a potential protective effect at higher loadings. At 75% diamond content, a pronounced decrease in stability is recorded (lowest T_d_ and T_d,max_ values), although the residual mass values align well with the theoretical diamond fraction, confirming the dominant contribution of the filler. The overall trend is similar to PLLA/MDP125 systems: higher diamond contents promote earlier degradation of the polymer due to enhanced thermal conductivity. However, this effect is not strictly linear and depends on the filler-to-matrix ratio [27]. Diamond’s exceptional thermal conductivity (10-fold enhancement in modified systems) generates localized thermal gradients affecting void formation through differential thermal contraction mechanisms. Surface functionalization reduces interfacial thermal resistance by eliminating air gaps and promoting intimate contact between diamond particles and polymer chains through chemical bonding. Enhanced thermal conductivity pathways enable controlled porosity development through uniform heat dissipation during matrix solidification, preventing irregular void formation from large-scale thermal gradients. Effective stress transfer through surface group-mediated interphase regions establishes local stress distribution networks controlling both pore nucleation density and growth kinetics. A detailed comparison of the properties of polymer-based composites loaded with diamond particles [28,29,30,31,32] is listed in Table S9.

Diamond particle incorporation into PLLA matrices induces heterogeneous stress concentrations, promoting agglomerate-mediated void nucleation and irregular pore development. Pristine diamond exhibits poor interfacial adhesion, generating localized stress concentrators that initiate cavitation toughening mechanisms through multiple cracking events and debonding at particle–polymer interfaces [9]. The agglomeration tendency creates preferential void coalescence sites, resulting in heterogeneous pore size distributions and compromised matrix integrity. Surface functionalization fundamentally alters pore formation pathways through enhanced interfacial compatibility mediated by specific surface functional groups. Diamond surfaces, typically hydroxylated through post-treatment with strong oxidants such as piranha solution, present reactive -OH groups that enable controlled chemical modification [10]. These hydroxyl functionalities serve as anchoring sites for coupling agents, establishing hydrogen bonding interactions between diamond surfaces and polymer matrices. Modified diamond particles create bridging structures through surface-grafted molecular chains that facilitate uniform stress distribution and controlled void nucleation, transitioning pore morphology from irregular agglomerate-associated voids to homogeneous porosity patterns with narrower size distributions. These surface groups prevent particle aggregation by providing steric stabilization and electrostatic repulsion, promoting uniform dispersion that directly influences void nucleation density and spatial distribution throughout the polymer matrix. Thermal behavior modifications significantly influence pore development through altered crystallization kinetics mediated by surface group interactions. Diamond incorporation reduces glass transition temperatures while increasing cold crystallization temperatures, indicating disrupted polymer chain mobility and modified segmental relaxation processes [16]. Surface functional groups enhance supramolecular matrix-filler interactions, creating kinetic barriers that promote amorphous region formation, which serve as preferential pore nucleation sites during processing.

## 4. Conclusions

The investigation demonstrated the successful synthesis and characterization of poly(L-lactic acid)-diamond composite foams with systematically tunable porosity properties through freeze-drying methodology. The incorporation of both 0.125 μm and 1.00 μm diameter microcrystalline diamond particles into the polymer matrix resulted in significant morphological and structural modifications without establishing chemical interactions between the constituent phases, as confirmed by infrared spectroscopy analysis. Morphological characterization through scanning electron microscopy revealed progressive reduction in surface complexity and development as diamond content increased. The polymer matrix exhibited characteristic wrinkled and coiled morphology, creating complex porous networks in neat polymer, which transitioned to more uniform diamond-layer coverage at higher concentrations (75 wt%), effectively forming polymer-in-diamond composites rather than diamond-in-polymer systems.

Porosity tunability emerged as a defining characteristic of these composite systems, with controllable porosity ranging from 11.4% to 32.8%, depending on diamond particle size and concentration. For 0.125 μm particle systems, porosity decreased systematically from 27.3% at 5 wt% to 11.4% at 75 wt%, while 1.00 μm particle systems demonstrated similar trends with anomalous behavior at low concentrations, exhibiting unexpectedly low porosity of 7% at 5 wt% loading. X-ray computed microtomography analysis revealed fundamental transitions from large-volume interconnected pores in neat polymer to numerous small-volume closed pores with increasing diamond content, fundamentally altering pore architecture and connectivity patterns throughout the composite structure.

Thermal characterization revealed complex interactions between diamond incorporation and polymer crystallization behavior. Differential scanning calorimetry analysis demonstrated decreased melting temperatures from 180 to 181 °C to 172 °C for 75% 0.125 μm particle loading and reduced melting enthalpies from approximately 46 J/g to 14.5 J/g, indicating modified crystalline structure formation and disrupted polymer chain organization. Conversely, thermogravimetric analysis showed reduced thermal stability due to enhanced heat transfer through high thermal conductivity diamond particles, accelerating polymer degradation at lower temperatures compared to neat polymer matrix.

Spectroscopic confirmation through Raman spectroscopy validated successful diamond incorporation via the characteristic diamond lattice vibration at 1332 cm^−1^, with intensity proportional to diamond content while maintaining polymer spectral integrity. The absence of new peaks in infrared spectra confirmed purely physical interactions, with observed peak intensity variations attributed to polymer polymorphism rather than chemical modification. These findings establish microcrystalline diamond–polymer composites as viable candidates for applications requiring biodegradable materials with engineered porosity control and enhanced thermal properties for tissue engineering scaffolds, thermal management systems, and specialized filtration technologies.

Economic prospects and commercial viability of PLLA-diamond composite foams present significant opportunities within expanding sustainable materials markets. The global PLLA market, valued at $1.25 billion in 2024 and projected to reach $4.22 billion by 2032, creates favorable conditions for commercialization. However, substantial economic barriers exist, with raw material costs ranging from $203–604/kg for 10% nanodiamond loading compared to $3–5/kg for baseline PLLA. Commercial viability requires strategic market positioning targeting high-value applications in biomedical devices, aerospace thermal management, and specialized filtration systems where performance advantages justify premium pricing. Initial market entry through niche applications with 2–5-year development timelines appears most promising, with broader industrial adoption contingent upon achieving cost reduction through manufacturing scale-up and process optimization to reach target pricing for mass market penetration. Large-scale fabrication requires advanced mixing technologies (twin-screw extrusion, ultrasonic processing) to achieve consistent diamond particle dispersion throughout the PLLA matrix. Surface modification via silanization of MDP particles presents a viable pathway to enhance interfacial bonding while preserving the demonstrated physical interactions and porosity control mechanisms. However, the specific porosity and crystallinity trends observed in PLLA-diamond systems must be validated across alternative polymer matrices, solvents, and post-processing conditions. Diamond’s unique thermal conductivity may induce fundamentally different crystallization kinetics and foam morphologies in other material systems, necessitating comprehensive process optimization for each polymer–diamond combination to ensure reproducible manufacturing outcomes.

## Figures and Tables

**Table 1:** Diamond microparticle types and parameters.

Sample	Type	Commercial Name	Median (D50) [µm]	Median Tolerance| [µm]	Upper Limit (D99) [µm]
MDP125	HPHT	MSY0-0.25	0.125	0.105–0.145	0.33
MDP1000	HPHT	MSY0.75-1.25	1.00	0.95–1.05	1.70

**Table 2:** Composition of fabricated PLLA/MDP samples.

Sample	PLLA Content [wt%]	MSY0-0.25 Content [wt%]	MSY0.75-1.25 Content [wt%]
PLLA	100	0	0
PLLA/MDP125-5	95	5	0
PLLA/MDP125-10	90	10	0
PLLA/MDP125-15	85	15	0
PLLA/MDP125-50	50	50	0
PLLA/MDP125-75	25	75	0
PLLA/MDP1000-5	95	0	5
PLLA/MDP1000- 10	90	0	10
PLLA/MDP1000-15	85	0	15
PLLA/MDP1000-50	50	0	50
PLLA/MDP1000-75	25	0	75

## Data Availability

The original contributions presented in this study are included in the article/Supplementary Material. Further inquiries can be directed to the corresponding author.

## Supplementary Materials

The following supporting information can be downloaded at: https://www.mdpi.com/article/10.3390/ma18194606/s1, Table S1: Coverage of polymer matrices calculated on the basis of SEM images for PLLA/MDP125 and PLLA/MDP1000; Table S2: Calculation of porosity for two reference samples (neat PLLA) and all diamond contents of MDP125 and MDP1000 in PLLA; Table S3: Porosity and density of PLLA/MDP125 composites; Table S4: Porosity and density for PLLA/MDP1000 composites; Table S5. Parameters for PLLA/MDP125 obtained from DSC (second heating scan); Table S6: Parameters for PLLA/MDP1000 obtained from DSC (second heating scan); Table S7: TGA parameters for PLLA/MDP125 foams; Table S8: TGA parameters for PLLA/MDP1000 foams; Table S9: Comparison of properties of diamond particles loaded polymer-based composites.

## References

[1] Zulkharnay R., May P.W. (2024). Applications of diamond films: A review. Funct. Diam..

[2] Field J.E. (2012). The mechanical and strength properties of diamond. Rep. Prog. Phys..

[3] Li Y., Yang L., Jiang X., Lu Y., Han C., Tang Y., Yang N. (2024). Diamond Nanostructures at Different Dimensions: Synthesis and Applications. Adv. Funct. Mater..

[4] Kowalewska E., Ficek M., Formela K., Zieliński A., Kunuku S., Sawczak M., Bogdanowicz R. (2022). Tailoring of Optical Properties of Methacrylate Resins Enriched by HPHT Microdiamond Particles. Nanomaterials.

[5] Rehman A., Houshyar S., Wang X. (2021). Nanodiamond-Based Fibrous Composites: A Review of Fabrication Methods, Properties, and Applications. ACS Appl. Nano Mater..

[6] Cai N., Dai Q., Wang Z., Luo X., Xue Y., Yu F. (2014). Preparation and Properties of Nanodiamond/Poly(Lactic Acid) Composite Nanofiber Scaffolds. Fibers Polym..

[7] Zhao Y.-Q., Lau K.-T., Kim J., Xu C.-L., Zhao D.-D., Li H.-L. (2010). Nanodiamond/Poly (Lactic Acid) Nanocomposites: Effect of Nanodiamond on Structure and Properties of Poly (Lactic Acid). Compos. Part B Eng..

[8] Zhang C., Ai M., Wu M., Rong J., Naito K., Yu X., Zhang Q. (2024). Effect of Curcumin-Modified Nanodiamonds on Properties of Eco-Friendly Polylactic Acid Composite Films. Colloids Surf. Physicochem. Eng. Asp..

[9] Han W., Wu M., Rong J., Zhang S., Zhang X., Zhao T., Yu X., Naito K., Zhang Q. (2023). Effect of Functionalized Nanodiamond on Properties of Polylactic Acid Eco-Friendly Composite Films. Diam. Relat. Mater..

[10] Su S.-H., Huang Y., Qu S., Liu W., Liu R., Li L. (2018). Microdiamond/PLA Composites with Enhanced Thermal Conductivity through Improving Filler/Matrix Interface Compatibility. Diam. Relat. Mater..

[11] Li F.N., Li Y., Bao H.W., Wang H.X., Ma F. (2023). Fabrication of Hydroxyl Terminated Diamond by High-Voltage Hydroxide Ion Treatments. Appl. Surf. Sci..

[12] Tsuji H., Aratani T., Takikawa H. (2013). Physical Properties, Crystallization, and Thermal/Hydrolytic Degradation of Poly(L-lactide)/Nano/Micro-Diamond Composites. Macromol. Mater. Eng..

[13] Zhang Y., Pan X., Bi M., Pan S., Zhang Z., Ma W., Wang X., Fu Y. (2023). Enhanced Separation Performance of Polyimide Ultrafiltration Membranes Embedded with Deep Eutectic Solvent-Coated Nanodiamonds. Ind. Eng. Chem. Res..

[14] Siddiqa A., Majid A., Saira F., Farooq S., Qureshi R., Qaisar S. (2023). Nanodiamond Embedded Polyaniline/Polyvinylidene Fluoride Nanocomposites as Microfiltration Membranes for Removal of Industrial Pollution. RSC Adv..

[15] Iqbal S., Rafique M.S., Iqbal N., Bashir S., Malarvili M.B., Anjum A.A. (2024). Development of Versatile, Thermally Stable, Flexible, UV-Resistant and Antibacterial Polyvinyl Alcohol-Nanodiamonds Composite for Efficient Food Packaging. Heliyon.

[16] Cieślik M., Susik A., Banasiak M., Bogdanowicz R., Formela K., Ryl J. (2023). Tailoring Diamondised Nanocarbon-Loaded Poly(Lactic Acid) Composites for Highly Electroactive Surfaces: Extrusion and Characterisation of Filaments for Improved 3D-Printed Surfaces. Microchim. Acta.

[17] Ficek M., Głowacki M.J., Gajewski K., Kunicki P., Gacka E., Sycz K., Mrózek M., Wojciechowski A.M., Gotszalk T.P., Gawlik W. (2021). Integration of Fluorescent, NV-Rich Nanodiamond Particles with AFM Cantilevers by Focused Ion Beam for Hybrid Optical and Micromechanical Devices. Coatings.

[18] Głowacki M.J., Ficek M., Sawczak M., Wcisło A., Bogdanowicz R. (2022). Fluorescence of Nanodiamond Cocktails: pH-Induced Effects through Interactions with Comestible Liquids. Food Chem..

[19] Szustakiewicz K., Gazińska M., Kryszak B., Grzymajło M., Pigłowski J., Wiglusz R.J., Okamoto M. (2019). The Influence of Hydroxyapatite Content on Properties of Poly(L-Lactide)/Hydroxyapatite Porous Scaffolds Obtained Using Thermal Induced Phase Separation Technique. Eur. Polym. J..

[20] Waheed S., Cabot J.M., Smejkal P., Farajikhah S., Sayyar S., Innis P.C., Beirne S., Barnsley G., Lewis T.W., Breadmore M.C. (2019). Three-Dimensional Printing of Abrasive, Hard, and Thermally Conductive Synthetic Microdiamond–Polymer Composite Using Low-Cost Fused Deposition Modeling Printer. ACS Appl. Mater. Interfaces.

[21] Alakrach A.M., Noriman N.Z., Dahham O.S., Hamzah R., Alsaadi M.A., Shayfull Z., Syed Idrus S.Z. (2018). Chemical and Hydrophobic Properties of PLA/HNTs-ZrO_2_Bionanocomposites. J. Phys. Conf. Ser..

[22] Dos Santos C.A.B., Ujčić A., Sobótka M., Jurczak P., Lach S., Rodziewicz-Motowidło S., Szustakiewicz K. (2023). Amber Extract as a Bio-additive to Poly(Lactic Acid) Films: Multimethod Analysis of Crystallinity and Stability. J. Vinyl Addit. Technol..

[23] Udvardi B., Kovács I.J., Fancsik T., Kónya P., Bátori M., Stercel F., Falus G., Szalai Z. (2017). Effects of Particle Size on the Attenuated Total Reflection Spectrum of Minerals. Appl. Spectrosc..

[24] Stehlik S., Mermoux M., Schummer B., Vanek O., Kolarova K., Stenclova P., Vlk A., Ledinsky M., Pfeifer R., Romanyuk O. (2021). Size Effects on Surface Chemistry and Raman Spectra of Sub-5 Nm Oxidized High-Pressure High-Temperature and Detonation Nanodiamonds. J. Phys. Chem. C.

[25] Tu J., Wang X., Liu B. (2024). Fragmentation Behavior of Diamond Particles with Different Particle Size and Ratio under High Pressure Compaction. Ceram. Int..

[26] Szustakiewicz K., Kryszak B., Gazińska M., Chęcmanowski J., Stępak B., Grzymajło M., Antończak A. (2020). The Effect of Selective Mineralization of PLLA in Simulated Body Fluid Induced by ArF Excimer Laser Irradiation: Tailored Composites with Potential in Bone Tissue Engineering. Compos. Sci. Technol..

[27] Zhang J., Wang J., Zhang G., Huo Z., Huang Z., Wu L. (2024). A Review of Diamond Synthesis, Modification Technology, and Cutting Tool Application in Ultra-Precision Machining. Mater. Des..

[28] Maitra U., Prasad K.E., Ramamurty U., Rao C.N.R. (2009). Mechanical Properties of Nanodiamond-Reinforced Polymer-Matrix Composites. Solid State Commun..

[29] Neitzel I., Mochalin V., Knoke I., Palmese G.R., Gogotsi Y. (2011). Mechanical Properties of Epoxy Composites with High Contents of Nanodiamond. Compos. Sci. Technol..

[30] Kalsoom U., Waheed S., Paull B. (2020). Fabrication of Humidity Sensor Using 3D Printable Polymer Composite Containing Boron-Doped Diamonds and LiCl. ACS Appl. Mater. Interfaces.

[31] Sun Y., Finne-Wistrand A., Waag T., Xing Z., Yassin M., Yamamoto A., Mustafa K., Steinmüller-Nethl D., Krueger A., Albertsson A. (2015). Reinforced Degradable Biocomposite by Homogenously Distributed Functionalized Nanodiamond Particles. Macromol. Mater. Eng..

[32] Behler K.D., Stravato A., Mochalin V., Korneva G., Yushin G., Gogotsi Y. (2009). Nanodiamond-Polymer Composite Fibers and Coatings. ACS Nano.

