# Supplementary material for: Engineered Porosity in Microcrystalline Diamond-Reinforced PLLA Composites: Effects of Particle Concentration on Thermal and Structural Properties
Source: Materials (Basel). 2025 Oct 4;18(19):4606. doi: 10.3390/ma18194606 (PMC12526470; doi:10.3390/ma18194606)
Supplement: Supplementary file 1 [file materials-18-04606-s001.zip › materials-3776321-supplementary.pdf]

Supplemental information to

Engineered Porosity in Microcrystalline Diamond-Reinforced PLLA Composites: Effects of Particle Concentration on Thermal and Structural Properties

Mateusz Ficek[1], Franciszek Skiba[1], Marcin Gnyba[1], Gabriel Strugała[2], Dominika Ferneza[3], Tomasz Seramak[4], Konrad Szustakiewicz[3] and Robert Bogdanowicz[1*]

[1] *Faculty of Electronics, Telecommunications, and Informatics, Gdańsk University of Technology, 11/12 Narutowicza St., 80-233 Gdańsk Poland*
[2] *Department of Materials Science and Technology, Institute of Manufacturing and Materials Technology, Faculty of Mechanical Engineering and Ship Technology, Gdańsk University of Technology, 80-233 Gdańsk, Poland*
[3] *Department of Polymer Engineering and Technology, Faculty of Chemistry, Wrocław University of Science and Technology (WUST), Wyb. Wyspiańskiego 27, Wrocław 50-370, Poland*
[4] *Department of Manufacturing and Production Engineering, Institute of Manufacturing and Materials Technology, Faculty of Mechanical Engineering and Ship Technology, Gdańsk University of Technology, 80-233 Gdańsk, Poland*
*Corresponding author: tel. +4858 347 1503, email: rbogdan@eti.pg.edu.pl (*R. Bogdanowicz*)

Table S1. Coverage of polymer matrices calculated on the basis of SEM images for PLLA/MDP125 and PLLA/MDP1000.

Sample	Surface coverage, [%]
PLLA/MDP125-5	8.59
PLLA/MDP125-10	4.30
PLLA/MDP125-15	9.85
PLLA/MDP125-50	70.69
PLLA/MDP125-75	91.00
PLLA/MDP1000-5	2.86
PLLA/MDP1000-10	5.87
PLLA/MDP1000-15	5.57
PLLA/MDP1000-50	19.07
PLLA/MDP1000-75	45.30

Table S2. Calculation of porosity for two reference samples (neat PLLA) and all diamond contents of MDP125 and MDP1000 in PLLA.

Sample	Volume of ROI	Volume of solid material in ROI	Volume of pores in ROI	Total porosity
	[mm³]	[mm³]	[mm³]	[%]
**PLLA**	108.5	82.1	26.5	24.4
**PLLA/MDP125-5**	108.6	78.9	29.6	27.3
**PLLA/MDP125-10**	108.5	90.9	17.6	16.2
**PLLA/MDP125-15**	108.5	91.3	17.1	15.8
**PLLA/MDP125-50**	108.5	91.5	17.0	15.7
**PLLA/MDP125-75**	108.5	96.1	12.3	11.4
**PLLA**	108.6	72.9	35.7	32.8
**PLLA/MDP1000-5**	108.3	100.7	7.6	7.0
**PLLA/MDP1000-10**	108.6	76.2	32.4	29.8
**PLLA/MDP1000-15**	108.4	92.3	16.1	14.9
**PLLA/MDP1000-50**	108.4	91.0	17.5	16.1
**PLLA/MDP1000-75**	108.5	91.5	16.9	15.6

Table S3. Porosity and density of PLLA/MDP125 composites.

Sample	Density, [g/cm^3]	Porosity, [%]
PLLA	0.050±0.007	96.0±0.5
PLLA/MDP125-5	0.052±0.005	96.0±0.4
PLLA/MDP125-10	0.056±0.002	95.8±0.1
PLLA/MDP125-15	0.054±0.005	96.1±0.4
PLLA/MDP125-50	0.078±0.008	95.8±0.4
PLLA/MDP125-75	0.143±0.006	94.1±0.2

Table S4. Porosity and density for PLLA/MDP1000 composites.

Sample	Density, [g/cm^3]	Porosity, [%]
PLLA	0.046±0.006	96.4± 0.5
PLLA/MDP1000-5	0.052±0.009	96.0 ±0.7
PLLA/MDP1000-10	0.058±0.004	95.6 ±0.3
PLLA/MDP1000-15	0.055±0.003	96.0 ±0.2
PLLA/MDP1000-50	0.095±0.007	95.5±0.4
PLLA/MDP1000-75	0.145±0.007	94.1±0.2

Table S5. Parameters for PLLA/MDP125 obtained from DSC (second heating scan).

Sample	T_g [°C]	T_{cc} [°C]	H_{cc} [J/g]	T_{m1} [°C]	T_{m2} [°C]	H_m [J/g]
PLLA	62.7	163.4	-4.32	180.8	-	46.38
PLLA/MDP125-5	50.1	165.3	-0.002	180.9	-	44.78
PLLA/MDP125-10	64.9	164.6	-0.48	181.3	-	41.83
PLLA/MDP125-15	65.9	-	-	180.4	-	41,28
PLLA/MDP125-50	63.2	-	-	175.9	178.7	45.52
PLLA/MDP125-75	56.8	-	-	171.9	-	58.64

Table S6. Parameters for PLLA/MDP1000 obtained from DSC (second heating scan).

Sample	T_g [°C]	T_{cc} [°C]	H_{cc} [J/g]	T_{m1} [°C]	T_{m2} [°C]	H_m [J/g]
PLLA	62.7	163.4	-4.32	180.8	-	46.38
PLLA/MDP1000-5	63.5	-	-	178.2	180.9	57.39
PLLA/MDP1000-10	62.0	156.9	-0.9	174.3	-	43.06
PLLA/MDP1000-15	61.8	155.3	-1.11	172.4	-	40.85
PLLA/MDP1000-50	63.7	-	-	176.4	180.6	56.08
PLLA/MDP1000-75	59.2	157.3	-0.89	175.2	-	26.56

Table S7. TGA parameters for PLLA/MDP125 foams.

Sample	$T_{-5\%}$ [°C]	T_d [°C]	$T_{d, max}$ [°C]	m_{580} [%]	m_{880} [%]
PLLA	342.1	353.7	369.0	0.68	0.62
PLLA/MDP125-5	340.9	353.0	368.7	3.72	3.64
PLLA/MDP125-10	344.0	353.7	369.1	8.45	8.25
PLLA/MDP125-15	344.9	354.6	369.4	15.78	15.15
PLLA/MDP125-50	324.9	345.9	371.0	49.55	48.84
PLLA/MDP125-75	314.7	325.2	357.4	73.81	72.77

Table S8. TGA parameters for PLLA/MDP1000 foams.

Sample	$T_{-5\%}$ [°C]	T_d [°C]	$T_{d, max}$ [°C]	m_{580} [%]	m_{880} [%]
PLLA	342.1	353.7	369.0	0.68	0.62

PLLA/MDP1000-5	333.1	349.2	366.9	5.59	5.49
PLLA/MDP1000-10	323.8	346.1	365.5	10.59	10.44
PLLA/MDP1000-15	324.3	343.8	365.4	14.93	14.84
PLLA/MDP1000-50	345.0	352.8	368.8	46.79	46.71
PLLA/MDP1000-75	328.3	335.0	363.9	74.53	74.33

Table S9. Comparison of properties of diamond particles loaded polymer-based composites.

	**Polymer matrix**	**Diamond characteristic**	**Performance**	**Reference**
**1**	PVA	5 nm, 0-0.6 wt%	Elastic modulus increase by 98.5% (from 0.67 to 1.33 GPa), and hardness increase by 78.6% (from 38.3 to 68.4 MPa) for the highest diamond content	[28]
**2**	Epoxy polymer	5 nm, 12-25 vol%	Elastic modulus increased 470% and hardness increased 300%. Improved scratch resistance and 25% thermal conductivity improvement	[29]
**3**	PLA	100 nm, 0-5 wt%	50.6% improvement of elastic modulus and 247.9% improvement of thermal conductivity. Increased tensile strength and elongation break	[9]
**4**	PLA	100 nm, 0-5 wt%	Elastic modulus increased from 1.29 to 2.29 GPa (177.5% improvement) T5% temperature increased by 47.9%	[8]
**5**	PANI/PVDF	50 nm, 0-5%	Increased water filtration efficiency	[14]
**6**	ABS	BDD 2-3 um, 60 wt%	Addition of LiCl to the composite resulted in fabrication of excellent humidity sensor	[30]
**7**	Poly(LLA-co_CL)	DND diamond, 1-5 0 wt%	Porosity of around 90%. Thanks to the modified particle surface no agglomeration occurred	[31]
**8**	PAN, PA	5nm, Up to 80 wt% for PAN Up to 40 wt% for PA	Fabrication of electrospun transparent fibres with improved mechanical properties: 4 times higher elastic modulus and 2 times higher hardness	[32]
**9**	PVA	5nm, 0-0.016 mg/ml	Fabrication of efficient UV filter with tensile strength increased 4 times	[15]
**10**	ABS	2-4 µm, 37.5-60 wt%	41.9% Improvement of elastic modulus and 5 times higher thermal conductivity	[20]
**11**	PI	DND diamond, 0-1 wt%	Hydrophilic modification of nanodiamond improved filtration properties of fabricated ultrafiltration membranes	[13]
**12**	PLLA	125 nm, 1µm, 0-75 wt%	Porosity decreased with addition of MDP125 and MDP1000 by reducing number of the single large-volume and increasing number of small closed pores	This work
